# A physical map of the heterozygous grapevine 'Cabernet Sauvignon' allows mapping candidate genes for disease resistance

**DOI:** 10.1186/1471-2229-8-66

**Published:** 2008-06-13

**Authors:** Marco Moroldo, Sophie Paillard, Raffaella Marconi, Legeai Fabrice, Aurelie Canaguier, Corinne Cruaud, Veronique De Berardinis, Cecile Guichard, Veronique Brunaud, Isabelle Le Clainche, Simone Scalabrin, Raffaele Testolin, Gabriele Di Gaspero, Michele Morgante, Anne-Francoise Adam-Blondon

**Affiliations:** 1 UMR de Génomique Végétale, INRA-CNRS-UEVE, 2, Rue Gaston Crémieux, CP5708, 91057 Evry Cedex, France; 2 UMR118, INRA-Agrocampus, University of Rennes, Amélioration des Plantes et Biotechnologies Végétales, F-35650 Le Rheu, France; 3 Dipartimento di Scienze Agrarie e Ambientali, University of Udine, via delle Scienze 208, 33100 Udine, Italy; 4 Unité de Recherche Génomique-Info, URGI, Tour Evry 2, 523, Place des Terrasses de l'Agora, 91034 Evry Cedex, France; 5 Gnoscope, 2, rue Gaston Crémieux, CP5706, 91057 Evry Cedex, France; 6 Dipartimento di Scienze Matematiche, University of Udine, via delle Scienze 208, 33100 Udine, Italy; 7 Istituto di Genomica Applicata, Parco Scientifico e Tecnologico Luigi Danieli, via Jacopo Linussio 51, 33100 Udine, Italy

## Abstract

**Background:**

Whole-genome physical maps facilitate genome sequencing, sequence assembly, mapping of candidate genes, and the design of targeted genetic markers. An automated protocol was used to construct a *Vitis vinifera *'Cabernet Sauvignon' physical map. The quality of the result was addressed with regard to the effect of high heterozygosity on the accuracy of contig assembly. Its usefulness for the genome-wide mapping of genes for disease resistance, which is an important trait for grapevine, was then assessed.

**Results:**

The physical map included 29,727 BAC clones assembled into 1,770 contigs, spanning 715,684 kbp, and corresponding to 1.5-fold the genome size. Map inflation was due to high heterozygosity, which caused either the separation of allelic BACs in two different contigs, or local mis-assembly in contigs containing BACs from the two haplotypes. Genetic markers anchored 395 contigs or 255,476 kbp to chromosomes. The fully automated assembly and anchorage procedures were validated by BAC-by-BAC blast of the end sequences against the grape genome sequence, unveiling 7.3% of chimerical contigs. The distribution across the physical map of candidate genes for non-host and host resistance, and for defence signalling pathways was then studied. NBS-LRR and RLK genes for host resistance were found in 424 contigs, 133 of them (32%) were assigned to chromosomes, on which they are mostly organised in clusters. Non-host and defence signalling genes were found in 99 contigs dispersed without a discernable pattern across the genome.

**Conclusion:**

Despite some limitations that interfere with the correct assembly of heterozygous clones into contigs, the 'Cabernet Sauvignon' physical map is a useful and reliable intermediary step between a genetic map and the genome sequence. This tool was successfully exploited for a quick mapping of complex families of genes, and it strengthened previous clues of co-localisation of major NBS-LRR clusters and disease resistance *loci *in grapevine.

## Background

Physical maps built from large-insert BAC clones and anchored to linkage maps assist sequence assembly in whole genome shotgun (WGS) sequencing projects [1], enable positional cloning of genes/QTLs and structural studies on gene families [2,3], and facilitate the isolation of homologous genes from model plants in heterozygous or polyploid species [4].

In plants, physical maps have been constructed for *Arabidopsis thaliana *[5], sorghum [6], rice [7], soybean [8], apple [9], black cottonwood [10], and grapevine [11]. Strategies based on BAC fingerprints detect overlaps among BAC clones for the development of physical maps. Briefly, BAC clones are digested with restriction enzymes and the fragments are separated by electrophoresis, producing a pattern of bands. The overlap between adjacent clones is identified by pairwise comparison of band profiles and calculation of the proportion of shared bands [12]. Technologies for producing BAC fingerprints have evolved rapidly, from using one to five restriction enzymes, and moving from agarose-based gel to sequencer-based electrophoresis to generate the profiles [13]. The methods that combine the use of several restriction enzymes and acrylamide gels or sequencers are usually referred to as High Information Content Fingerprinting (HICF), because they have allowed a dramatic increase in the sensitivity of the process. Since all of these methodologies were not applied to the same biological materials, the performances are not homogeneously comparable, and the debate on the advantages and disadvantages of the different protocols still persists [14]. For instance, it appears that the five-enzyme restriction protocol developed by [13] leads to a higher error rate per fingerprint, but provides the highest sensitivity compared with alternative techniques based on two or three-enzyme restrictions [15]. In the present work, the five-enzyme restriction protocol was adopted because of its higher sensitivity and throughput.

The grapevine genome has recently disclosed two peculiarities: grapes are highly heterozygous and they have descended from an ancient hexaploid ancestor [1,16,17]. The polyploid origin of the grapevine genome was revealed by whole proteome comparison [1] but was undetectable using STS markers or nucleotide alignments. Thus homeologous regions are not expected to hamper the construction of a physical map of the grapevine genome, as their respective fingerprints are substantially different. In turn, heterozygosity is likely to affect the correct assembly of BAC fingerprints, as it did in the DNA assembly of *Ciona savignyi *[18]. This aspect was addressed in poplar by [10], but was somewhat neglected in grapevine [11] and in apple [9]. Here, a thorough analysis of the effects of heterozygosity on physical map construction is presented that unveils contig features that were not previously described in poplar. It is also shown that the final map is an effective tool for mapping candidate genes for agronomic traits like disease resistance, as well as for developing new genetic markers.

Strengthening the resistance to diseases is one of the major objectives in grapevine breeding [19]. Two types of defence can be categorized in plants, based on the width of the host range. Non-host resistance is effective across an entire plant *taxon *against all isolates of a pathogen. Host resistance, the second type of resistance, is exerted at a genotype-to-genotype level: only some of the genotypes of a plant *taxon *to which a pathogen has adapted are resistant to any or all pathogenic strains. This classification agrees well with that based on the type of mechanisms and genes involved. Pre-invasion barriers and reactions triggered by pathogen-associated molecular patterns (PAMPs), called PAMP-triggered immunity (PTI), disrupt the potential ability of a pathogen to attack a plant. Overall, the concepts of non-host resistance and PTI overlap. Host resistance is a second line of defence towards pathogens that have gained the capability of suppressing basal or PAMP-triggered resistance. Host resistance can be either complete or partial. The mechanisms underlying complete resistance are similar across many types of plants. Complete host resistance is conferred by R proteins that recognise pathogen effectors/suppressors or modifications of their cellular targets (effector triggered immunity, ETI). R genes are mostly arrayed in clusters, a physical organisation that generates new variants at a rate higher than in any other class of genes [20,21]. The links between ETI, PTI, complete host, and non-host resistance were modelled by [22]. Both PTI and ETI rely first on pathogen recognition carried out by receptors, consisting of transmembrane proteins for PTI [23,24], and cytoplasmic proteins with a nucleotide-binding site and a leucine-rich repeat domain (NBS-LRR) or receptor-like kinases (RLK) for ETI [25]. These two sides of the immune system are connected by proteins of the downstream signalling pathways, such as SGT1 and RAR1 [26-28], and by gene products of the salicilic acid (SA), jasmonic acid (JA), ethylene (ET), and MAPK cascade pathways [29].

Non-host resistance is the outcome of a heterogeneous set of genes, and the proteins they specify, that are implicated in pathogen accessibility/inaccessibility (*i.e*. lipase-like *EDS1*, synthaxin-like *PEN1*, *etc*.), cytoskeletal rearrangements and protein turnover (*i.e. SGT1*), PAMP-triggered responses, and synthesis of toxic metabolites [30-32]. The location of their homologues in the grapevine genome was recently established using information from the grapevine genome sequence [1,17], which was unknown when this study began. In contrast, the genes triggering complete host resistance all conform to a few classes of receptor-coding genes that are functionally similar. Clues on the size and the genomic organisation of R gene families in grape were given by genetic map data [2,33,34] and by a survey in the draft genome sequence [17].

In this paper, we present (1) the assembly of a 'Cabernet Sauvignon' physical map based on restriction enzyme BAC fingerprinting, (2) the anchorage of the physical contigs on the meiotic linkage maps, (3) the use of this map for the placement of candidate genes for disease resistance, and (4) the alignment between the physical location of resistance gene analogues and phenotypic *loci *for pathogen resistance based on bridging markers. How the high heterozygosity in the 'Cabernet Sauvignon' genome has shaped some features of the physical map is also studied in detail and discussed.

## Results

### Construction of the 'Cabernet Sauvignon' physical map

The 44,544 BAC clones of the 'Cabernet Sauvignon' BAC library, which represent about 12.3 genome equivalents [35], were fingerprinted using the method described by [11]. Raw data were trimmed for background signals, vector peaks, chloroplastic clones, intra-plate contaminations, and chimerical clones. A total of 30,828 high-quality fingerprints (70%) were used to assemble a map, using FPC 8.2 and following the iterative approach proposed by [36]. In the final version of the map 29,727 clones were ordered in contigs, while 1,111 clones (3.6%) remained as singletons [see Table 1]. The physical map includes 1,770 contigs comprised of 650,622 unique bands spanning 715,684 kbp in physical length, a size that is 1.5-fold larger than the actual genome size. The contigs were made up of 17.4 clones on average and had an average physical size of 404 kbp. The assembly included 2,982 (10%) questionable clones (Qs), which are clones of uncertain position. These clones were found in 577 (32.6%) questionable contigs.

**Table 1:** Features of the 'Cabernet Sauvignon' physical map.

Number of clones fingerprinted	44,544
Number of clones used for map assembly	30,828
Number of singletons	1,111
Number of contigs	1,770
> 200 clones	5
101–200 clones	10
51–100 clones	85
26–50 clones	238
11–25 clones	520
3–10 clones	702
2 clones	210
Unique bands of the contigs	650,622
Physical length of the contigs (Mbp)	715,684
Number of Q clones	2,982
Number of Q contigs	577

The average band size (1.1 kbp) was estimated by dividing the average insert size of the BAC clones by the average number of bands per clone used for fingerprinting. This value was used to estimate the physical length of the contigs.

### Integration of physical and genetic maps and assessment of the quality of the physical map

The alignment between the physical and the linkage maps was initiated using the SSR markers genetically mapped by [37], and preliminarily reported by [38]. Primer pairs were used for PCR screening of 18,432 BAC clones, corresponding to 6 genome equivalents, which identified 1,833 positive BAC clones, corresponding to 5 clones per marker on average. Out of the 368 initial markers, 24 were not useful for the integration because they localised on BAC clones that did not yield useful fingerprints (15 cases) or on singleton clones (9 cases). The remaining 344 SSR markers anchored 335 contigs covering 220.2 Mbp and corresponding to 30.8% of the total size of the physical map [see Additional file 1]. Out of these 335 contigs, a unique genetic position could be assigned to 312 contigs that correspond to 190 Mbp [see Additional file 2, contigs in yellow boxes]. The location of the other contigs remained ambiguous, because the markers physically localised on a given contig were genetically assigned to more than one linkage group [see Additional file 2, contigs in grey boxes]. Out of the 312 genetically anchored contigs, 82 (26.3%) were anchored by two or more markers and covered 64.1 Mbp, whereas the other 230 contigs were anchored by only one marker and covered 127.7 Mbp. The average size of anchored contigs was 656.7 kbp, whereas the average size of non-anchored contigs was 344.3 kbp. The map is available at [39].

Contigs containing genes relevant to this work and not tagged by the markers of the reference *Vitis *map were anchored by SSR markers positioned in other genetic maps [34], by newly developed SSR markers, and by SSCP markers [see Additional file 1]. These markers were projected onto the reference *Vitis *map by map alignment with common markers, and are reported underlined and in italics [see Additional file 2]. They allowed the anchoring of 60 additional contigs, expanding the size of the anchored map to 255 Mbp. This corresponds to 35.7% of the total map size [see Table 2].

**Table 2:** Integration of physical and genetic maps.

*Linkage group*	*No. of contigs*	*Coverage (kbp)*	*Average contig size (kbp)*
1	20	14,895	745
2	14	9,752	696
3	13	5,600	431
4	15	12,289	820
5	19	9,348	492
6	18	10,318	573
7	19	12,065	635
8	20	11,714	585
9	17	11,309	664
10	19	12,199	642
11	16	9,579	598
12	24	14,678	612
13	35	18,337	524
14	27	16,804	623
15	14	7,820	558
16	13	7,522	579
17	16	10,841	678
18	29	19,612	675
19	19	8,664	455
Unknown*	28	32,141	1,147
No. of anchored contigs	395	255,476	647
No. of non anchored contigs	1,375	460,208	334

*Total*	1,770	715,684	405

The markers used for integration consisted of 344 microsatellites from the reference map of [37], 45 microsatellites present in other genetic maps, newly developed markers from contigs that carried genes relevant to this study, and 23 SSCP markers for NBS-LRR genes [34]. The physical length of the contigs was estimated based on an average band size of 1.1 kbp.

* Not assigned to a linkage group because two or more markers placed on the physical contig mapped to two or more linkage groups.

The quality of the physical map was tested using two types of checks: first, an estimate was obtained for the percentage of chimerical contigs, that is BAC clones from different genomic regions spuriously assembled in the same contig, and second, an assay on the reliability of BAC clone order within physical contigs was performed.

Two independent tests were applied to estimate the percentage of chimerical contigs. The first test examined 118 contigs on which two or more genetically mapped markers were localised. In 28 contigs (23.7%), inconsistency was observed between the genetic and physical position of the markers. Markers with independent segregation were found physically linked in the same contig. In order to determine whether it originated from erroneous genetic data or from an inherent genomic complexity that mixed up BACs in the contig assembly, these 28 contigs were further examined. For each marker, the genomic location and the presence of unique annealing sites for the corresponding primer pair were validated and confirmed using the PN40024 genome sequence [1]. Thus, it was concluded that the chimaeras were not caused by marker duplication or misplacing. Little evidence was found that it could be due to homeology; only contig 1769 contained two markers pointing to two homeologous regions of the grapevine genome. For the second test, the BAC clones were aligned to the PN40024 genome sequence through their unique BAC End Sequences (BES). Out of 846 contigs made up of at least two BACs that aligned to the sequence, 61 (7.3%) appeared to be potentially chimerical. As this value of chimerism is much lower than the one predicted using molecular markers, the BAC alignment to the genome sequence of the 28 marker-chimerical contigs was examined in detail. For 17 contigs (61%), the inconsistency was due to a single BAC clone found to match one molecular marker, and in 10 out of these 17 cases, the same molecular marker also matched BAC clones of other contigs. It is thus likely that in these 17 cases, the overall construction of the contig was correct except for a local mis-insertion of a single alien BAC. In the other 11 cases, the chimaeras predicted by molecular markers were confirmed by the location of the BES on the genome sequence. Some automatic steps of the assembling procedure were reconsidered. The BAC clones to which the questionable molecular markers belong were not joined to the contig by the merging steps during the iterative assembly [15], but they had mixed up earlier during the automatic assembly and the first DQing steps. Indeed, 10 out of these 11 contigs showed a suspiciously high percentage of Q clones (20% to 67%) compared with the average value (9.8%). These 11 contigs were rebuilt using a Sulston's cutoff score of 1e-60. In only 2 out of 11 cases (18%) the physical linkage in the chimerical contig could be broken. It can be argued that these types of contigs hide a biological complexity which can not be properly handled using automatic procedures.

The second study to assess the quality of the physical map consisted of a detailed analysis of how BAC clones are ordered relative to one another within contigs in two arbitrarily selected genomic regions. The first region was at the top of LG 5 between markers VVMD27 and UDV-060, and was covered by two physical contigs. The second region comprised 3 contigs in the interval between the markers VMC9G4 and VVIB09 on LG 17. The SSR markers were re-scored on all the BAC clones included in the corresponding region [see Additional file 3]. The BAC clones were then aligned to the PN40024 genome sequence using their BES.

**Figure 1:**
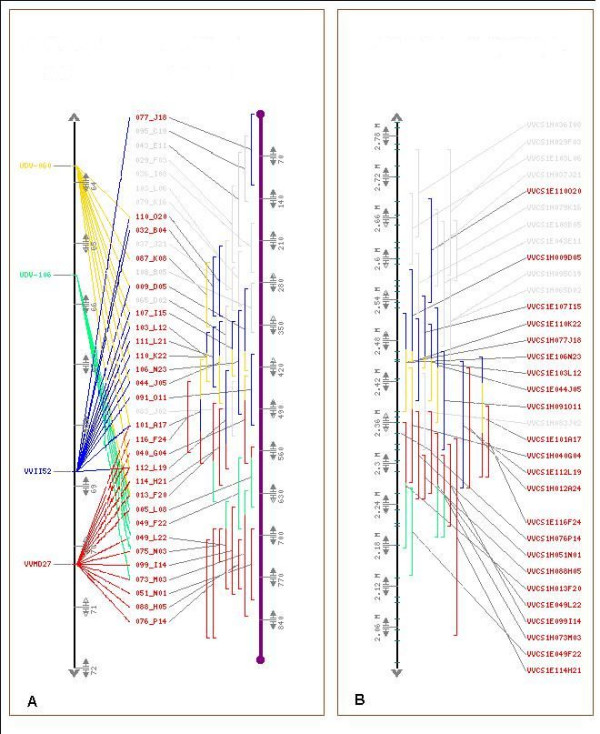
**Alignment of the contig 207 along the reference genetic map (A) and the 8.4× genome assembly (B)**. The genetic markers are indicated on the left of LG 5 (vertical black bar on the left; distances in cM) with different colours. The BAC clones, indicated by vertical bars on the right of **A **and **B**, are positioned into the physical contig according to FPC calculations (**A**) or according to the alignment of their end sequences on the 8.4× genome assembly (Vertical black bar on the left of **B; **distances in Mbp). Their respective colours correspond to the genetic marker they carry.

Two main problems were observed. The first one was an incorrect order of the BAC clones within a contig, producing apparent duplications of *loci *in the physical map. An example of this effect is shown for the contig 207 [see Figure 1A]. The VVMD27 genetic marker was physically localised on two separate groups of BAC clones as if they belonged to a duplicated region. The VVMD27 primer pair aligned to a unique position on the genome assembly and the end sequences of the BAC clones that carried VVMD27 aligned to the same single genomic region as well [see Figure 1B], showing that the apparent duplication in the physical contig corresponded to a single *locus *in the genome sequence. The same pattern of apparent intra-contig duplications could also be seen in a less obvious manner for VVII52 and UDV-060 [see Figure 1A]. The second effect of heterozygosity was the assembly of the BAC clones corresponding to the two different haplotypes into separate contigs, which was clearly represented in the LG 17. In this case, no artifactual intra-contig duplication was observed, but two separate contigs containing allelic BAC clones of the marker VVIB09 were identified [see Additional file 3]. The largest one (contig 1676) was made of 32 BAC clones, while the other one (contig 2388) was made of only 3 BAC clones and corresponded to the alternate haplotype of the region targeted by VVIB09 in the contig 1676. The unique match of the VVIB09 primer pair and of the BES of all its BAC clones on the PN40024 genome sequence confirmed that contig 1676 and contig 2388 were allelic. Both effects led to an inflation of the size of the physical map and may explain why the total length of the 'Cabernet Sauvignon' physical map is 1.5 fold the size of the grapevine genome.

### Identification of disease resistance gene homologues in the BAC clones

Three different classes of resistance genes (non-host, host, and signalling related resistance genes) were searched for by PCR screening of 18,432 BAC clones and *in silico *screening of 77,237 BAC-end sequences [see Table 3].

**Table 3:** Number of BAC clones and BAC contigs found to be anchored by homologues of different categories of genes involved in resistance to pathogens.

	PCR screening		Blast search of BES		Total	
	
	Number of BAC clones	Number of contigs	Number of BAC clones	Number of contigs	Number of BAC clones	Number of contigs
**Host**	248	93 (30)	1317	399 (111)	1527	424 (115)
NBS-LRR	234	88 (29)	985	315 (87)	1196	346 (95)
RLK	14	6 (1)	370	148 (56)	370	148 (56)
**Non-host and signalling**	144	45 (33)	112	62 (28)	249	99 (57)
**Total of non redundant BAC clones or contigs**	392	136 (61)	1411	414 (125)	1757	484 (154)

The number of contigs anchored to the genetic map is indicated in brackets.

Sixty-one out of 66 primer pairs tested for NBS-LRRs identified 234 non-redundant BAC clones among which 26 were positive for two to three different primer pairs. Then 182 grape ESTs analogous to NBS-LRRs matched 985 BES in a tBlastX search. The corresponding BAC clones were checked for redundancy with those identified by PCR: 962 BAC clones were unique, raising the number of BAC clones containing NBS-LRRs to 1,196. Most of these new BAC clones were identified by EST queries that span the LRR region (791) whereas queries spanning the NBS region mostly identified new BAC clones from the half of the library not included in the 6× sub-library screened by PCR (171). Four primer pairs targeting the RLK gene family identified 14 BAC clones. The BES were queried using 27 RLK gene fragments, leading to the identification of 356 additional BAC clones, which raised the total number to 370.

Thirty primer pairs targeting genes involved in non-host resistance and signalling pathways [see Additional file 4] allowed the identification of 144 BAC clones, with an average of 4.8 BAC clones per primer pair. Only the primer pair targeting *SGT1 *did not amplify any BAC clone. The grapevine ESTs used for primer design were also used for blast search of BES and retrieved 112 BAC clones. By combining PCR screening and *in silico *searches, the number of non redundant BAC clones containing homologues for non-host resistance and defence signalling genes was raised to 249.

### Physical organisation of NBS-LRR and RLK genes for host resistance

**Figure 2:**
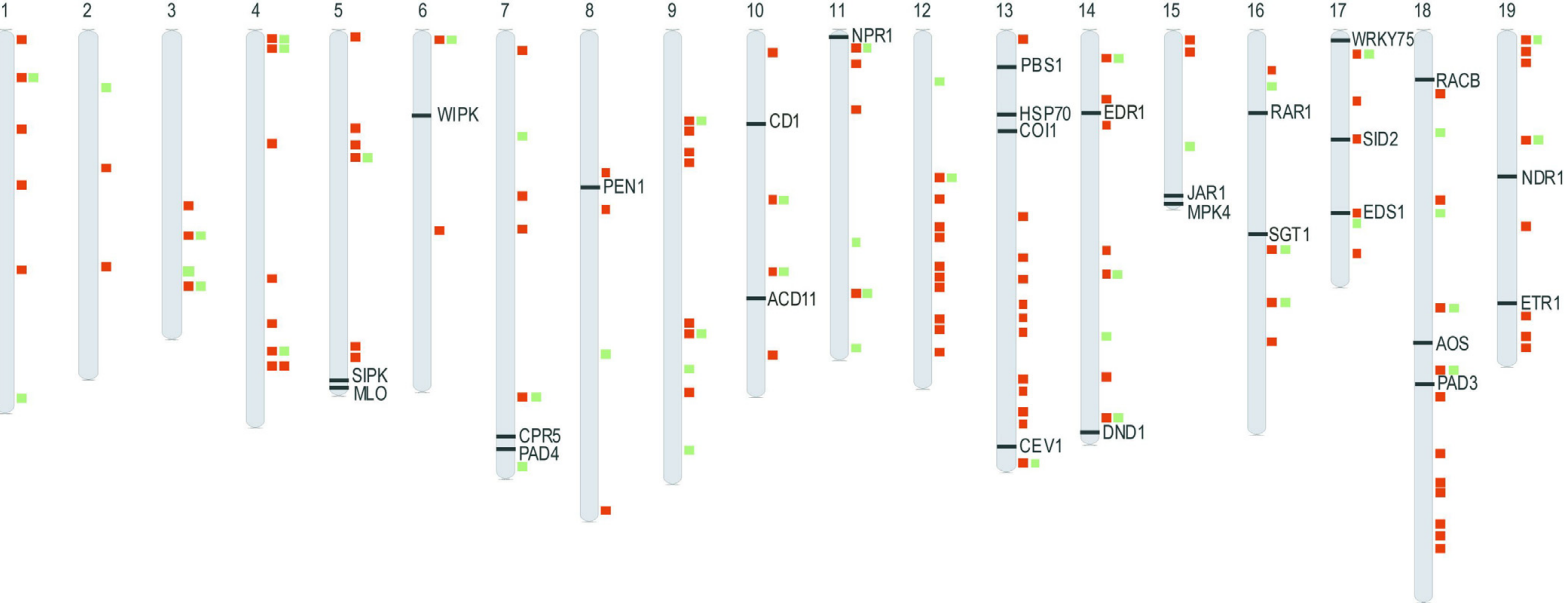
**Distribution of grapevine homologues to non-host, host, and defence signalling genes**. The 19 grapevine chromosomes were drawn according to the orientation and the genetic distances of the *Vitis *reference map [37]. Gene localisation was inferred based on the integration of the physical and genetic data. The position of the 27 homologues to non-host and disease-resistance signalling genes (black horizontal ticks) are given only for the physical contigs of 'Cabernet Sauvignon' identified by PCR screening. The position of the grapevine analogues to NBS-LRR class resistance genes are indicated with red boxes and the RLK class resistance genes with green boxes.

Out of all 1,527 BAC clones that contained host resistance genes, 1,097 BAC clones (72%) were assembled into 424 contigs among which 346 (17% of all contigs of the physical map) contained NBS-LRR sequences. Out of these, 94 were assigned to linkage groups by the reference markers of [37]. Twenty-one additional contigs were anchored by other SSRs, raising the total number to 115, which corresponds to 27% [see Figure 2 and Additional file 2, red boxes]. As much as 16% of the contigs containing analogues to host resistance genes were positive for both NBS-LRR and RLK classes. In particular, RLKs were found in contigs that also contained NBS-LRRs in 47% of the cases [see Table 3].

**Figure 3:**
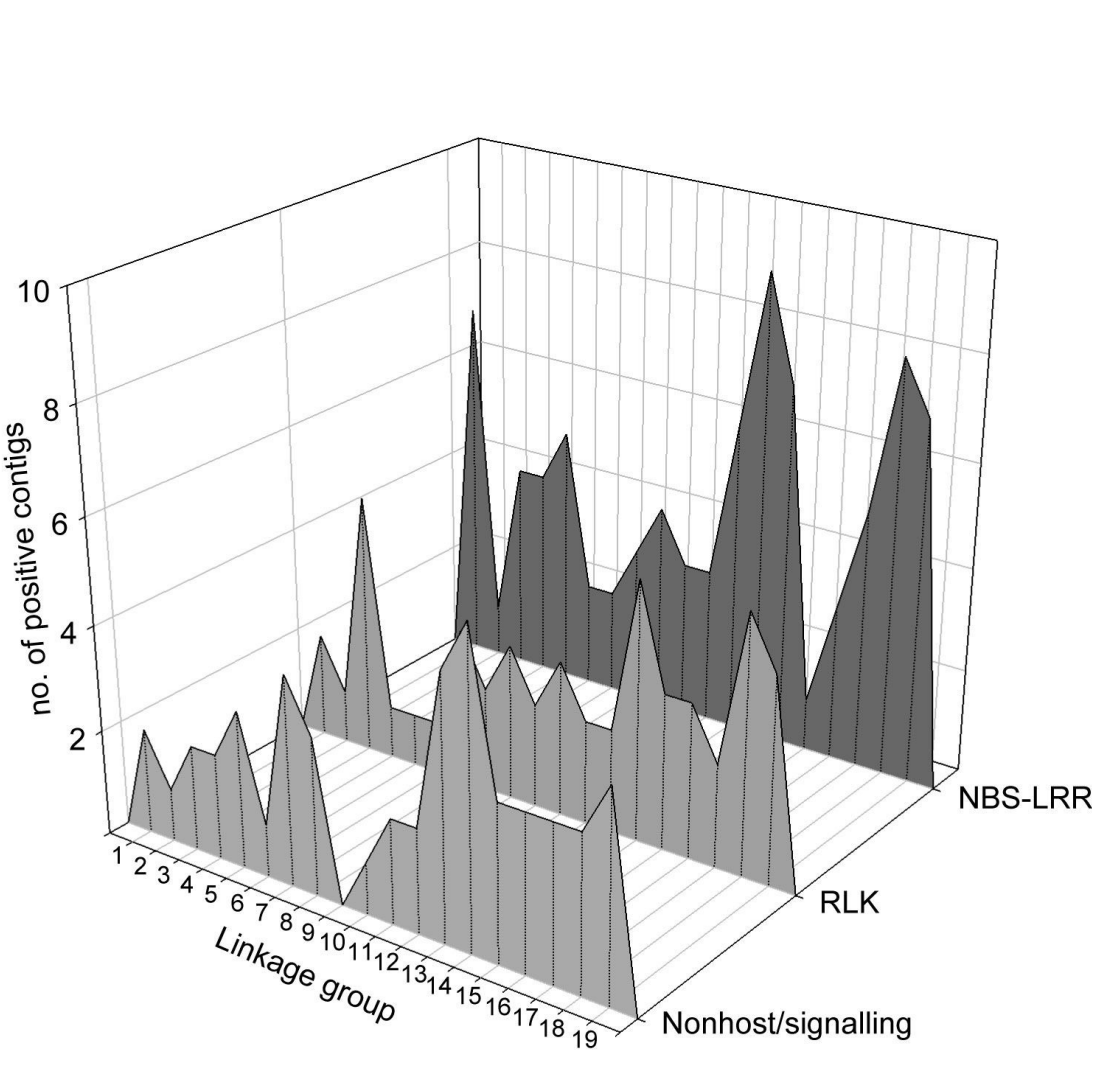
**Abundance of non-host, host, and defence signalling genes**. The number of BAC contigs containing resistance related genes and anchored on each linkage group is reported for non-host and signalling genes and for two families of host resistance genes, NBS-LRR, and Pto-like kinases.

The contigs containing NBS-LRRs were present on all chromosomes with a skewed distribution within each chromosome, whereas the contigs containing RLKs were more evenly dispersed across the genome [see Figure 2 and Additional file 2, green boxes]. LGs 12, 13, and 18 were the richest in NBS-LRRs, and LG 14 scored the highest number of contigs containing RLKs [see Figure 3].

### Physical localisation of non-host resistance gene homologues and genes for SA, JA, ET, and MAPK cascade signalling pathways

Out of 249 BAC clones that contained non-host resistance and signalling genes, 167 (67%) were assembled into 99 contigs. Out of these, 38 were anchored by the reference markers of [37] and 19 by newly developed SSRs, raising the total number of anchored contigs to 57 for this category [see Additional file 2, blue boxes]. Contigs containing genes for non-host resistance and signalling pathways were evenly dispersed across the genome, even though contigs containing sequences homologous to genes of signalling pathways were more frequently found on LGs 14, 15, and 19 [see Figure 2]. Contigs positive for this category of genes contained host resistance gene analogues in 39% of the cases.

When the genome survey was restricted to the 144 BAC clones on which homologues of non-host and signalling genes were identified by PCR, these BAC clones were assembled into 45 contigs, corresponding to 1.5 contigs per gene on average [see Additional file 5]. At least one copy of each of the 30 genes studied, except *SGT1*, was represented in the 45 contigs. For ten genes, two contigs per gene were found which might correspond to allelic contigs. This hypothesis is corroborated by the observation that the corresponding primer pair matched a single *locus *in the PN40024 genome sequence. Three or four contigs were found for the genes *MPK4*, *PBS1*, *ACD11*, and *RACB*.

Eleven genes were assigned to a physical contig which was anchored by the reference markers of [37]. SSR markers, linked to 19 unassigned genes, were newly developed [see Additional file 5]. For two genes, microsatellites could be developed using the physical assembly. In these cases, microsatellites were found in the BES of another BAC from the same contig. For 17 genes, microsatellites were developed using the PN40024 genome sequence. The primer pairs of the gene, previously used to physically localise the gene in the BAC clones, were used for a BlastN search of the genome sequence. When perfect matches for the primer sites were found, a search was performed for microsatellites in the assembled sequence within a 40-kbp interval around the gene. These new markers were mapped using either of the two mapping populations reported in [34] and projected onto the reference map of [37].

Four genes (*HSP90*, *NHO1*, *RIN4*, and *ACD11*) were physically assigned to contigs but the corresponding contigs could not be genetically anchored by any available marker. Two genes, *JAR1 *and *ACD11*, were physically assigned to contigs that remained genetically unmapped, but the primer pairs of each gene had a perfect match on two scaffolds of the genome sequence attributed to LG 15 and LG 10, respectively. *SGT1 *was not physically assigned to any BAC clone but a unique sequence of the gene was found on the genome assembly, and a new SSR marker found within a 3-kbp interval around the gene (SGT1_SSR) was mapped to LG 16.

A genomic position of the highest identical analogue could thus be assigned to 27 out of the initial 30 non-host and resistance signalling genes that were initially searched for in the BAC library [see Additional file 2]. The position of each gene was validated by the perfect match of corresponding primer pair on the anchored scaffolds of the PN40024 genome sequence.

Genes causing interference with cell wall penetration of biotrophs, like *PEN1 *and *MLO *in *Arabidopsis *and barley [32,40], account for resistance against powdery mildews, a disease important also to grape. The grape homologue of *PEN1 *is located on LG 8, and the homologue of the broad spectrum resistance gene *MLO *is located on LG 5. Various plant genes, like lipase-like *EDS1*, glycerol kinase *NHO1*, and ubiquitin ligase-associated protein *SGT1*, can halt infection after a non-host pathogen has penetrated [41-43]. Grapevine homologues of *EDS1 *and *SGT1 *were localised to LG 17 and 16, respectively, however, the homologue of *NHO1 *could not be localised using this approach. SA, JA/ET, and MAPK cascades are components of the signalling networks that are required for mounting inducible defences through specific routes to the non-host and the host defence. We localised the grape homologues of SA synthesis and signalling genes, such as *SID2 *(LG17), *NDR1 *(LG19), *EDS1 *(LG17), and *NPR1 *(LG11); of JA synthesis and signalling, such as *AOS *(LG18), *COI1 *(LG13), and *JAR1 *(LG15); ET-insensitive *ETR1 *(LG19); and MAP kinases, such as *SIPK *(LG5), *WIPK *(LG 6), *EDR1 *(LG14), and *MPK4 *(LG15). Complete resistance, triggered by many R genes, also requires the interacting proteins RAR1 (on LG16) and SGT1, found on LG16 as well [27,43]. Among other genes involved in the activation of the HR, the homologue of the cyclic nucleotide-gated ion channel gene *DND1*, associated with a host resistance that is uncoupled from cell death in *Arabidopsis*, was located at the bottom of LG14 in the grape genome. *RACB*, the grape homologue of *OsRac1 *that acts as a component of the programmed cell death (PCD) pathway in rice, was located on LG18. The homologue of Accelerated Cell Death 11 (ACD11) was located on LG10.

## Discussion

The 'Cabernet Sauvignon' physical map described here is the second one constructed for a highly heterozygous grapevine cultivar, while the first one was based on 'Pinot Noir' [11,44]. Both of them were assembled using the same fingerprinting method. A common feature of both maps is that the total length of the contigs is much larger than the estimated size of the grape genome (1.5–1.6 fold). In the 'Cabernet Sauvignon' map, this expansion was mainly attributed to the effects of heterozygosity. 'Cabernet Sauvignon' is an offspring of the cross between 'Cabernet franc' and 'Sauvignon Blanc' [45]. Genotyping with SSR markers showed a high level of heterozygosity for this cultivar [46]. In 'Pinot Noir', it was recently shown that an important part of the sequence variation is due to insertion/deletion events, and that the frequency of SNPs is uneven [17]. The same features were observed in a preliminary experiment that compared two haplotype sequences in 'Cabernet Sauvignon' over two different genomic regions encompassing 182 kbp (unpublished data). These features affect the banding pattern of the fingerprints produced from two allelic regions, and were thus expected to hamper the proper assembly of the corresponding BAC clones. The use of a set of BAC-anchored molecular markers with a unique position on the reference genome sequence, and checks of the alignment of the BAC clones using their BES on the same genome sequence proved that heterozygosity could lead to either a patchy ordering of heterozygous clones within a contig or to a separation of 'allelic' clones in two separate contigs. Both phenomena explain the observed 1.5-fold expansion of the physical map. Indeed, while 55% of the single-locus markers were found on allelic BAC clones that belonged to the same contig, the remaining 45% anchored two or more contigs.

These limitations, which cause difficulties in the proper assembly of BAC clones, are a common feature of physical maps produced for genomes of heterozygous species. Up to now, only three fingerprinting-based physical maps have been assembled for heterozygous plants other than grape: *Prunus *[47], apple [9], and black cottonwood [10]. The peach map was constructed using the same fingerprinting method as in grapevine, but other differences impair the comparison of the two assemblies. First, mapping in peach is still ongoing, and the clones fingerprinted so far, which represent 4.3× genome equivalents, are biased towards the expressed regions of the genome [48]. Second, two BAC libraries were used in peach, one obtained from a diploid genotype and the other from a haploid one, which could have attenuated the effect of heterozygosity. The black cottonwood and the apple maps show a 1.2-fold expansion of the genome size [9,10]. This value is lower compared to the ones found in grapevine, which may be somewhat explained by the use of agarose fingerprinting for producing the black cottonwood and the apple maps. According to [36], the confounding effect of bands that correspond to restricted repetitive elements, and the differences in fragment size caused by insertions/deletions of a few bases are likely to be more neutral in agarose fingerprinting than in sequencer-based methods, due to the larger size of the analysed fragments. In poplar, when aligning BES to the genome sequence, it was observed that two physical contigs frequently stuck to the same interval over the sequence [10], suggesting that separation of allelic contigs occurred as it did in the grapevine physical map. If a single haplotype was counted for each of the contig pairs that co-aligned over the sequence, then the overall estimation of the size of the physical map showed almost no expansion [10]. Finally, the black cottonwood map indicated that the whole genome duplication that occurred in the Salicaceae lineage impacted the physical map far less than the heterozygosity did [10]. If this holds true for a recent genome doubling, the effect of homeology in grapevine and other eudicots that share an ancient hexaploid origin should be even more negligible.

A validation of contig assembly of the 'Cabernet Sauvignon' map, based on independent controls from genetic markers and the PN40024 genome sequence, led us to estimate that 7.3% to 9.3% of the contigs built by FPC are chimaeras. Until now, the percentage of chimerical contigs in physical maps have been estimated in only few cases. A contig assembly error of 5% was reported in the channel catfish physical map [49], which was also built using the [13] method. The maize map, which was constructed with a three-colour based HICF method, showed 4% of false joins [50]. The percentage of chimaeras observed in the present work is close to this range. Physical chimerism may arise from common fingerprint bands corresponding to repetitive sequences or large-scale duplications shared by physically unlinked BAC clones. The ways in which transposable elements might interfere with WGS assembly were argued in rice [51], and the same arguments may also apply to misplacing of BAC clones in physical contigs of the grape physical map.

Knowing all of these limitations, we showed however that a physical map can be a useful complement to a genetic map to localize a set of candidate genes for agronomical traits. The physical map has been used to produce an inventory of genes, anchored to the chromosomes, potentially involved in resistance against pathogens. An overview of the genomic distribution of the physical contigs positive for different classes of genes is given [see Figure 2 and Figure 3]. This picture is very close to the analysis of the same gene families based on the sequence draft of 'Pinot Noir' [17], confirming the reliability of the physical mapping approach.

Based on the locations found for homologues of non-host resistance or signalling pathways, none of them reveal a positional candidate that's position corresponds with a known QTL for disease resistance [2,52-54]. By contrast, several physical contigs containing NBS-LRR genes were tagged by markers that are linked to major QTLs or genes for disease resistance reported in literature [2,34,52-54]. In most of these cases, the segregation of resistance suggested that several *loci *might contribute to the trait, but the *loci *tagged by the markers, that now correspond to contigs rich in NBS-LRRs, accounted for the largest effect [53]. Due to the difficulty in handling quantitative trait studies in a controlled environment for a perennial woody plant like grapevine, most of the experiments were carried out with small segregating populations, which allowed a reliable detection of only one or a few QTLs with the strongest effect. Increasing the sensitivity and resolution of QTL analysis may provide further information on the position of additional *loci *that contribute to the remaining part of phenotypic variance. Our work also provides markers that could improve the marker density of genetic maps in these regions and that could be used for marker assisted selection in breeding programs.

## Conclusion

A *Vitis vinifera *'Cabernet Sauvignon' physical map was constructed and a large set of candidate genes for pathogen resistances were anchored on it. Two main aspects were addressed which could be useful for further projects in the field of genomics.

First, the paper focused on the effects of high levels of heterozygosity on fingerprint-based physical maps. It was showed that an appropriate automated protocol could produce a proper assembly by reducing the impact of this potentially hampering factor.

Second, the map appeared to be a useful and reliable intermediary step between a genetic map and the genome sequence for the positioning of candidate genes. It allowed the quick mapping of complex families of genes, and strengthened previous clues of co-localisation of major NBS-LRR clusters and disease resistance *loci*.

## Methods

### BAC fingerprint-based assembly of the physical map

A BAC library of *Vitis vinifera *'Cabernet Sauvignon' was used for the construction of the physical map. It contained 44,544 clones with a mean insert size of 142 kbp representing about 12.3 genome equivalents [35,38]. A total of 77,237 BAC end sequences (BES) with a mean size of 671 bp were obtained from sequencing both ends of 44,544 BAC clones, and were retained after quality check [38]. These sequences randomly covered approximately 0.1 × of the grape genome. This library has been adopted as one of the reference genomic resources by the International Grape Genome Program network [55] and BAC clones are freely available upon request [56].

BAC clones were fingerprinted following the protocol published by [13] and adapted to grape as preliminarily reported by [11]. Briefly, DNA was isolated from each BAC clone, digested with four rare cutter endonucleases (*Eco*RI, *Bam*HI, *Nde*I, and *Xba*I) and a frequent cutter (*Hae*III). Fragments were labelled with the SNaPshot labelling kit (Applied Biosystems, Foster City, CA), purified using genCLEAN plates (Genetix, St James, NY, USA) and re-suspended in formamide. Fragments were separated by capillary electrophoresis on an ABI3730 automated sequencer (Applied Biosystems, Foster City, CA) and sized using the Genescan LIZ-500 internal size standard. The electrochromatograms were analyzed using GeneMapper 3.5 (Applied Biosystems, Foster City, CA). An output text file containing data of area, height, and size of each peak was generated and edited using a homemade perl script. The peaks corresponding to the background, to the vector, and the peaks shorter than 75 bp or longer than 500 bp were removed. Intra-plate contaminations and possible chimaeras were inspected and discarded by three checks. A first set was identified with the GenoProfiler package [57]. Second, all the clones that yielded more than 210, 230, and 250 peaks for inserts of 120, 140, and 160 kbp average size, respectively, were removed. Third, the BES of neighbouring clones were compared. If two or more neighbour clones presented a high identity for both BES (> 95% identity over > 95% of the length of the shortest BES in the pairwise alignment), the BAC clones harbouring the redundant BES were removed using a home made perl script. When only one BES was available for a given BAC clone and this BES was highly similar to a BES of a neighbour clone, the physical map was examined to check if those two clones were buried. If that was the case, the clone with only one BES available was removed.

Trimmed data were assembled using the software FPC 8.2 [58]. The tolerance was set at 0.4 and the first automatic assembly was performed under highly stringent conditions using a Sulston score of 1e-40. The contigs containing more than 10% of Q clones were split using the DQing option, with three rounds of analysis at progressively lower cut-off of 1e-45, 1e-50, and 1e-55. The contigs were then end-merged at a cut-off of 1e-35. Subsequently, the assembly was carried out following an iterative strategy [50] with alternate steps of end-merging and DQing, using progressively less stringent cut-off for end-merging of 1e-30, 1e-25, and 1e-20. After the last merging, singletons were inserted into the contigs using a cut-off of 1e-30.

The BAC assembly was validated based on the information of the molecular markers placed on each contig, and edited manually in three steps. First, each contig that included BAC clones with unlinked genetic markers was tentatively broken by re-assembling at a more stringent cut-off. Second, if the contig remained putatively chimerical at the most stringent cut-off of 1e-30, the primer pairs of the markers were blasted against the reference genome 8.4× assembly of *V. vinifera *'PN40024' [1] to assess the number of possible annealing sites and to confirm the chromosomal location. When the discrepancy between the genetic and the physical map was confirmed for single copy markers, the corresponding physical contig was considered chimerical. Finally, contigs containing BAC clones associated with genetically linked markers, were tested for merging at a less stringent cutoff of 1e-15.

### Analysis of BAC-end sequences

Chloroplastic contamination of the library was assessed using the complete chloroplast genome of *Vitis vinifera *(Embl accession number DQ424856, [59]) as a query for BlastN search. A BES was considered a chloroplastic sequence when a > 95% identity over > 100 bp was found by BlastN or when > 95% identity over 33–133 amino acids and > 80% identity over > 133 amino acids was found by tBlastX.

BES were masked for repetitive elements and then aligned to the PN40024 genome sequence through a Blat analysis (90% of identity on 80% of length, less than 5 hits) as reported in [1, Supplementary data]. The results were then filtered using homemade perl scripts, and a BAC clone was considered as aligned to the genome sequence only if both paired ends matched at a distance less than 300 kbp and with a consistent orientation [1, Supplementary data]. Physical contigs were filtered and a subset that met the following requirements was used for the validation. First, all the contigs made up of less than 3 BACs were discarded. Second, the contigs made up of 3 BACs that aligned to 2 or 3 different linkage groups were also discarded. A total of 846 physical contigs passed these two steps of trimming. Then, a contig was considered as chimeric if at least two BACs anchored onto a linkage group and if at least two other BACs anchored onto another linkage group.

### Physical localisation of markers and genes on the BAC clones

Primer pairs for microsatellite markers present in the genetic map of [37] and for genes relevant to this study were scored on BAC pools according to the protocol described by [38]. Microsatellite markers present in the reference genetic map were used to integrate the physical map and the genetic map, randomly across the genome.

The primers used for PCR screening of the BAC library for non-host, host, and signalling resistance genes are described in [Additional file 5] and were developed as follows. We first selected from *Arabidopsis thaliana *and *Nicotiana benthamiana *30 genes with proven functions in the above mentioned categories, and the corresponding proteins were downloaded from NCBI [see Additional file 5]. The amino acid sequences were used for tBLASTn search of the grape ESTs at the TIGR and NCBI databases as of September 1, 2006. Grape ESTs were found for all of the genes, and the EST showing the highest identity with the corresponding gene was retained. Selected ESTs were then compared by BLASTn against the 6× genome assembly of PN40024 available at that time to deduce the corresponding gene model. PCR primers were preferentially designed on a single exon, and over two sequence arrays without SNPs between the EST and the PN40024 sequence [see Additional file 5]. In addition, a tBlastN analysis was performed on the BES and the results were parsed with the following: E value < 1e-04 for all the queries excepted for those corresponding to large multigene families like *EDR1*, *MPK4*, *SIPK*, and *WIPK*, where the E value < 1e-20, and *PBS1 *where the E value < 1e-50.

The primers used for screening the BAC library for the NBS-LRR and RLK genes were described in [34]: 33 primers (series rgVamu and rgVrip) were originally developed from *Vitis amurensis *and *Vitis riparia*, 26 primers (series GLP and MHD) were designed on a *Muscadinia rotundifolia *× *Vitis vinifera *hybrid, 7 primers (series rgVvin and UDV-) were from *Vitis vinifera *'Cabernet Sauvignon', and four primers were designed on grape sequences with the highest similarity to the *Pto *(tomato) and *Xa21 *(rice) resistance genes (stkVa008, stkVa036, stkVa043, and stkVr011).

We also used 182 grapevine sequences for NBS-LRR proteins available at NCBI in October 2004 as queries for tBLASTx of the BES. Of these, 131 queries were gene fragments spanning the NBS domain and already presented in [34] GenBank accession no. AY427077–AY427135, AY427152–AY427194, AF369813–AF369837, AF365879–AF365881,, and AF365851 were ESTs that mostly covered the 3'-end of the LRR domain. The BES were also queried using 27 RLK gene fragments (AY427136–AY427151 and AY427195–AY427205).

### Development of additional SSR markers in genomic regions potentially involved in disease resistance

Some contigs containing candidate genes for resistance to pathogens were a ssigned to chromosomes by the SSR markers of the reference genetic map. A search for microsatellites was performed in the BES of all BAC clones included in these non-assigned contigs with a modified version of Sputnik [60], and used for the design of contig-specific SSR markers. When no useful SSR was found in the neighbour BES, the primer pairs of the gene previously used to physically localise the gene in the BAC clones were used for a BlastN search in the 6× assembly of PN40024. If a unique and perfect match was found with a distance between the primer sites compatible with the amplicon size obtained from the BAC clones, a search for additional SSRs was performed in sequence contigs of PN40024 within a 40-kbp interval around the gene [see Additional file 5]. The new markers were genetically mapped in the progeny 'Chardonnay' × 'Bianca' and 'Cabernet Sauvignon' × hybrid '20/3' [34]. Segregation data were added to the previous dataset as described in [34]. Marker positions were projected on the *Vitis *reference map by map alignment using common markers.

## Abbreviations

BAC: Bacterial Artificial Chromosome; BES: BAC End Sequence; ETI: Effector Triggered Immunity; HICF: High Information Content Fingerprinting; JA: Jasmonic Acid; MAPK: Mitogen Activated Protein Kinase; NBS/LRR: Nucleotide Binding Site/Leucine Rich Repeat; PAMP: Pathogen Associated Molecular Patterns; PTI: Pathogen Triggered Immunity; QTL: Quantitative Trait Loci; RLK: Receptor Like Kinase; SA: salicilic Acid.

## Authors' contributions

MaM physically mapped non-host and defence signalling genes and developed new SSR markers for contig anchorage, integrated the results from all contributors, and drafted the manuscript. SP constructed the physical map with the help of ILC, AC, CC, and VDB, realised the preliminary analysis of BES with LF, and participated in drafting the manuscript. RM screened the BAC library for host resistance genes. AC and ILC screened the BAC library for reference SSR markers. LF wrote the perl scripts for the BES analysis and maps visualisation. CG and VB developed databases for the following of experiments and the storage of the results. GDG and RT implemented genetic maps with new markers, participated in the interpretation of the results and in drafting the manuscript. SS and MiM contributed to map assembly. A–FA–B conceived this study, coordinated the construction of the physical map and finalised the manuscript. All authors read and approved the final manuscript.

## Supplementary Material

Additional file 1 This is a spreadsheet which lists all the physical contigs anchored by genetic markers and the origin of the markers, whether they had been previously used for the reference genetic map of [37] or if they were newly developed in the frame of the present work. Click here for file

Additional file 2 This is a figure which shows in a graphical way the integration between the genetic and physical map of grapevine. For each chromosome, all the mapped genetic markers are listed on its left side, while all the integrated physical contigs are listed on its right side. Furthermore, all the anchored resistance genes are indicated within boxes and a colour code is used to indicate both their functional category and the way in which they were mapped (i.e. by BAC pooling or by Blast of the BES). Click here for file

Additional file 3 This is a spreadsheet which summarizes the results of the analysis performed on two genomic regions to assess the effects of heterozygosity on the physical map. The left panel reports the results relative to the region between the markers UDV-060 and VVMD27 (on LG5), while the right panel reports the results relative to the region between the markers VMC9G4 and VVIB09 (on LG17). Both of the panels list all the contigs included in the two regions and for each contig, all the BACs are reported as well. Then, for each single BAC clone, the results of the PCR tests carried out with the above mentioned molecular markers are detailed. Click here for file

Additional file 4 This is a table which lists all the candidate genes for non-host resistance and for defence signalling. Several pieces of information are given for each gene, like its biochemical role, the NCBI accession number of the gene chosen from the model species, the TIGR accession number of the corresponding grapevine EST and the PCR primers used for BAC pooling. Click here for file

Additional file 5 This is a spreadsheet showing the results of the mapping of the candidate genes for non-host resistance and for defence signalling. Several pieces of information are reported for each gene, like the physical contigs onto which it was found and the newly developed SSR primers used to anchor the contigs (if any) on the genetic map. Click here for file
