# Supplementary figures and images for: A physical map of the heterozygous grapevine 'Cabernet Sauvignon' allows mapping candidate genes for disease resistance
Source: BMC Plant Biol. 2008 Jun 13;8:66. doi: 10.1186/1471-2229-8-66 (PMC2442077; doi:10.1186/1471-2229-8-66)

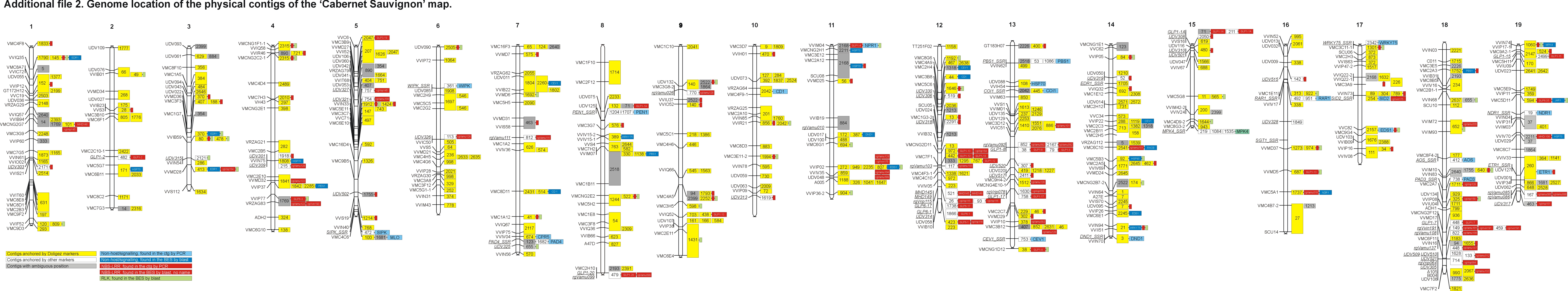

Supplement: Additional file 2 — This is a figure which shows in a graphical way the integration between the genetic and physical map of grapevine. For each chromosome, all the mapped genetic markers are listed on its left side, while all the integrated physical contigs are listed on its right side. Furthermore, all the anchored resistance genes are indicated within boxes and a colour code is used to indicate both their functional category and the way in which they were mapped (i.e. by BAC pooling or by Blast of the BES). [file 1471-2229-8-66-S2.jpeg]
